# Parental help-seeking behaviour for, and care of, a sick or injured child during the COVID-19 pandemic: a European online survey

**DOI:** 10.1186/s12913-023-09371-1

**Published:** 2023-04-25

**Authors:** Chantal D. Tan, Silvia Bressan, Rachel Carter, Mia Hylén, Inger Kristensson, Monica Lakhanpaul, Santiago Mintegi, Henriette A. Moll, Sarah Neill

**Affiliations:** 1 Erasmus MC – Sophia, Department of General Paediatrics, Rotterdam, The Netherlands; 2 Department of Woman’s and Child’s Health, University of Padova, Padua, Italy; 3 Faculty of Health, University of Plymouth, Plymouth, UK; 4 Department of Intensive and Perioperative Care, Skane University Hospital, Malmö, Sweden; 5 Department of Health Sciences, Faculty of Medicine, Lund University, Lund, Sweden; 6 UCL Great Ormond Street Institute of Child Health University College London, UCL, London, UK; 7 Cruces University Hospital, Biocruces Bizkaia Health Research Institute, University of the Basque Country, Bilbao, Basque Country Spain

**Keywords:** COVID-19, Children, Paediatrics, Pandemic, Parental help-seeking, Survey

## Abstract

**Background:**

Globally, the COVID-19 pandemic had a huge impact on patients and healthcare systems. A decline in paediatric visits to healthcare settings was observed, which might have been due to lower incidence of injury and infectious illness, changes in healthcare services and parental concern. The aim of our study was to examine parental experiences of help-seeking for, and care of, a sick or injured child during COVID-19 lockdown periods in five European countries with different healthcare systems in place.

**Methods:**

An online survey for parents with a child with any kind or illness of injury during COVID-19 lockdowns was circulated through social media in five European countries: Italy, Spain, Sweden, the Netherlands, and the United Kingdom. Parents living in one of these countries with self-identification of a sick or injured child during COVID-19 lockdown periods were eligible to fill in the survey. Descriptive statistics were used for the level of restrictions per country, children’s characteristics, family characteristics and reported help-seeking behaviour of parents prior to the lockdown and their real experience during the lockdown. The free text data was subjected to thematic analysis.

**Results:**

The survey was fully completed by 598 parents, ranging from 50 to 198 parents per country, during varying lockdown periods from March 2020 until May 2022. Parents who completed the survey were not deterred from seeking medical help for their sick or injured child during the COVID-19 pandemic. This finding was comparable in five European countries with different healthcare systems in place. Thematic analysis identified three main areas: parental experiences of access to healthcare, changes in parents’ help-seeking behaviours for a sick or injured child during lockdowns, and the impact of caring for a sick or injured child during the lockdowns. Parents reported limited access to non-urgent care services and were anxious about either their child or themselves catching COVID-19.

**Conclusion:**

This insight into parental perspectives of help-seeking behaviour and care for a sick or injured child during COVID-19 lockdowns could inform future strategies to improve access to healthcare, and to provide parents with adequate information concerning when and where to seek help and support during pandemics.

**Supplementary Information:**

The online version contains supplementary material available at 10.1186/s12913-023-09371-1.

## Introduction

The Coronavirus Disease 2019 (COVID-19) caused by the SARS-CoV-2 virus initially started in Wuhan, China in December 2019. First there was uncertainty concerning the infectivity and disease course, but soon it was considered as a fast-spreading infectious disease with a large range of clinical symptoms affecting the whole world and in particular its healthcare system [1]. Countries declared national public health emergencies and restrictions were taken to minimize the transmission, such as the stay-at-home advice and closing of schools. However, restrictions differed per country ranging from a very strict lockdown in Spain to more voluntary measures in Sweden [2, 3].

Globally, there was a large decline in paediatric visits to healthcare services during the COVID lockdown periods with reductions in attendances in Europe ranging from 31 to 85% [2, 4–7]. This might be explained by a lower incidence of injuries and infectious illness due to the lockdowns, changes in healthcare services and parental concern leading to changes in help-seeking behaviour. However, providing adequate healthcare is important during a pandemic, especially for children who are a vulnerable patient group. Several studies have shown that delayed presentation to healthcare services has led to severe illness and harm in children [8–10].

For this reason, it is important to evaluate the consequences of changes in healthcare services and parental perceptions of help-seeking behaviour for their sick or injured child during the COVID-19 pandemic. This could influence service design and delivery during periods of high demand at times other than a pandemic, such as improving access to healthcare. A survey of parents’ experiences with a sick or injured child during lockdown was first set out in the United Kingdom (UK) as a starting point, and extended to the Netherlands focusing on children with comorbidity [11, 12]. The aim of this study is to provide insight into the help-seeking behaviour and care for a sick or injured child from the parental perspective during the COVID-19 pandemic in five European countries with different healthcare systems and changes in healthcare services due to the COVID-19 pandemic. Additionally, we aimed to go into more depth on parental experiences during the COVID-19 pandemic by using parental quotes in the qualitative part of the survey.

## Methods

### Study design

This study is a collaboration between five European countries with the UK as the lead setting. Healthcare professionals at the University of Plymouth and University College London developed an online survey to assess parental help-seeking behaviour for, and care of, a sick or injured child during the COVID-19 lockdown periods. The survey mostly consisted of multiple-choice questions, but parents were also able to give more detailed information in open text fields (Additional file 1). This survey was translated into the native language of the participating countries and adapted to their own healthcare system. Informed consent of the participating parents was obtained and the study was approved by the ethics committees of all participating settings: United Kingdom (Ethics committee of University of Plymouth’s Faculty of Heath, Ref 2020–2216), The Netherlands (Ethics committee of Erasmus Medical Center, MEC-2020-0627), Spain (Ethics Committee of the Basque Country, code PI2021052), Italy (Ethics Committee of the University Hospital of Padova, prot n 0016770/21), Sweden (Swedish Ethical Review Authority, Ref. 2021–04947). All methods were performed in accordance with the relevant guidelines and regulations.

### Study population and setting

Parents living in one of the five European countries; Italy, Spain, Sweden, the Netherlands, and the UK, with self-identification of a sick or injured child during the COVID-19 pandemic were eligible to fill in the online survey. The introduction text of the online survey states that all responses are anonymous and parents were obliged to tick a box to confirm they have read the introductory information and that they want to fill in the survey (Additional file 1). The period in which the survey was actively disseminated, the lockdown periods and the level of restrictions per country are shown in Table 1. Parents who did not have an injured or sick child or did not live in the country of the launched survey were excluded from this study.

**Table 1:** Survey period and level of restrictions per country

	Active survey period	Lockdown periods	Level of restrictions during lockdown
**Italy**	30 March – 30 September 2021	First Lockdown: 9 March – 18 May 2020, then variation across regions according to the risk based on the surveillance system data	They varied depending on the Italian region. The first lockdown was very restrictive, with schools being closed and all the other non-essential activities as well.
**Spain**	30 March – 1 May 2021	First lockdown: 14 March – 21 June 2020 Restrictions between 25 October 2020–9 May 2021	Schools and universities closed, many professions working from home, limited access to Face-to-Face attendance in Primary Care.
**Sweden**	26 November 2021–12 May 2022	No lockdown, variable recommendations and restrictions between 10 March 2020 and 9 February 2022	Restrictions and recommendations according to the level of infection, stated by the government. Elementary schools stayed open, but for universities distance learning was recommended and large gatherings were prohibited. No outdoor activity restrictions and shops stayed open. Social distancing recommended.
**The Netherlands**	19 October – 19 December 2020 and 16 January – 16 February 2021	First lockdown: 23 March – 1 June 2020 Second lockdown: 15 December 2020–16 February 2021 Variable restrictions between lockdowns and until March 2022	Schools were closed, contact professions were prohibited, no group formation, social distancing.
**United Kingdom**	7 May – 23 June 2020	First lockdown: 7 May – 23 June 2020 Second lockdown: 5 November – 2 December 2020 Third lockdown: 6 January – 29 March 2021 Variable restrictions between lockdowns and after the third lockdown until 18th March 2022	Schools were closed, non-essential shops closed, contact professions prohibited, outdoor activity restricted to 30 min exercise a day.

### Data collection

The survey was circulated online using different platforms: SNAP survey, RedCap and Google Forms. Parents were recruited using social media including Facebook, Twitter, LinkedIn, Instagram and WhatsApp. Virtual snowball sampling was used to disseminate the survey further by reposting an advertising text and the link to the survey on social media. Data collected included children’s characteristics (age, gender, presenting symptoms, chronic condition or recurring illness, hospital admission), family characteristics (living area, access to technology), and parents’ response to their sick or injured child and their experiences. The two main questions were (1) whether parents would have sought help for the same medical problem of their sick or injured child before the lockdown, and (2) whether parents did seek help for the medical problem of their sick or injured child during the lockdown. Other questions concerned the impact of changes in health services on the perceived severity of illness and the treatment the child received.

### Data analysis

Data was analysed using SPSS software V.25.0 and thematic analysis was performed for the free text data using NVivo 12. Parents who answered the two main questions on help-seeking behaviour before and during the lockdown, were included for analyses. Descriptive statistics were performed for the children’s characteristics, family characteristics, and parents’ response to their sick or injured child before and during the COVID-19 lockdown, stratified by country. For the qualitative thematic analysis themes were identified from the free-text responses using Braun and Clarke’s methodology [13]. Responses to the following questions within the survey provided the qualitative data:

- Have the changes to health services during the Stay Home period affected how ill your child has been? If yes, please tell us how.
- Have the changes to health services during the Stay Home period affected any treatment your child received? If yes, please tell us how.
- What, if anything else, would you like to tell the project team?

## Results

### Children’s and family characteristics

Our study population consisted of 598 parents who completed the survey on the two main questions, ranging from 59 parents in Sweden to 198 parents in the UK. Table 2 shows the children’s characteristics stratified by country. The majority of the children were in the age group of five to twelve years and were boys. The presenting symptoms varied with pain as the most frequently presenting symptom. The number of children with a chronic condition and the number of children admitted varied per country.

**Table 2:** Children’s characteristics stratified by country

	Italy *N* = 72	Spain *N* = 164	Sweden *N* = 59	The Netherlands *N* = 105	United Kingdom *N* = 198	Overall *N* = 598
**Age** (years)						
< 1	11 (15)	23 (14)	9 (15)	13 (12)	12 (6)	68 (11)
1 < 2	12 (17)	36 (22)	6 (10)	13 (12)	17 (9)	84 (14)
2 < 5	22 (31)	45 (27)	16 (27)	15 (14)	37 (19)	135 (23)
5 < 12	21 (29)	48 (29)	14 (24)	43 (41)	99 (50)	225 (38)
12 < 16	5 (7)	7 (4)	10 (17)	14 (13)	26 (13)	62 (10)
16 < 18	1(1)	5 (3)	4 (7)	7 (7)	7 (4)	24 (4)
**Gender** (boys)	42 (58)	87 (53)	31 (53)	61 (58)	104 (53)	325 (54)
**Presenting symptom**^a^						
Skin and appearance	8 (11)	26 (16)	12 (20)	22 (21)	40 (20)	108 (18)
Breathing difficulties	7 (10)	28 (17)	20 (34)	25 (24)	36 (18)	116 (19)
Body temperature	18 (25)	35 (21)	20 (34)	16 (15)	26 (13)	115 (19)
Dehydration	12 (17)	17 (10)	25 (42)	21 (20)	36 (18)	111 (19)
Pain	27 (38)	78 (48)	32 (54)	47 (45)	107 (54)	291 (49)
Change of behaviour	20 (28)	33 (20)	16 (27)	42 (40)	59 (30)	170 (28)
Injury	27 (38)	42 (26)	16 (27)	23 (22)	55 (56)	163 (27)
Other	6 (8)	49 (30)	14 (24)	55 (52)	72 (36)	196 (33)
**Chronic condition or recurring illness**	14 (19)	29 (18)	8 (14)	51 (49)	51 (26)	153 (26)
**Admission**	Unknown^b^	8 (5)	21 (36)	37 (35)	24 (12)	90 (15)

Absolute numbers and percentages (%) are shown

^a^Possible to have more than one presenting symptom

^b^No information on hospital admission, 47% (34/72) of the visits were at hospital facility level

Family characteristics stratified by country are shown in Table 3. Most of the families lived in urban areas (range 47–94%) and a minority lived in rural areas. The majority have smart phones and a laptop, desk computer or tablet with unlimited WiFi access.

**Table 3:** Family characteristics stratified by country

	Italy^a^ *N* = 72	Spain^a^ *N* = 164	Sweden *N* = 59	The Netherlands *N* = 105	United Kingdom *N* = 198
**Description of living area (urban)**	42 (58)	145 (88)	28 (47)	99 (94)	126 (64)
**Smart phone**	52 (72)	159 (97)	49 (83)	105 (100)	195 (98)
**Laptop/desk computer/tablet**	49 (68)	153 (93)	46 (78)	100 (95)	191 (96)
**Unlimited WiFi access**	43 (60)	144 (88)	46 (78)	99 (94)	188 (95)
**All of the children were staying at home when the illness/injury happened during lockdown**	37 (51)	137 (84)	21 (36)	80 (76)	186 (94)

Absolute numbers and percentages (%) are shown

^a^The percentages are lower for these countries because the results include incomplete responses

**Fig. 1:**
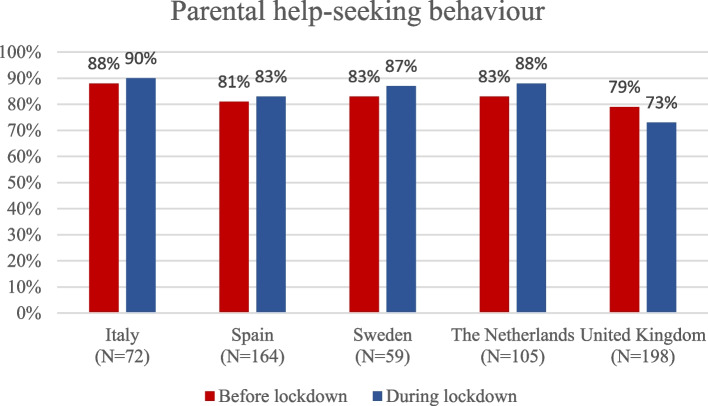
Parental help-seeking behaviour before and during lockdown stratified by country

Parental help-seeking behaviour for the same medical problem before and during lockdown was comparable in all countries as shown in Fig. 1. The percentage of help-seeking during lockdown compared to prior to the lockdown varied between 2% and 6% across countries, with only the UK reporting a lower percentage of help-seeking during the lockdown compared to before the lockdown.

### Parents’ experience of help-seeking for, and care of, a sick or injured child during lockdowns

Thematic analysis identified three main areas where the pandemic lockdowns influenced parents’ experience of seeking help for, and care of, a sick or injured child: Access to healthcare; Parents’ help-seeking behaviours; and the impact on parents of caring for a sick or injured child during lockdowns.

#### Access to healthcare for a sick or injured child during COVID-19 lockdowns

Parents in all five countries reported limited access to non-urgent care services, resulting in delays in initial consultations, a lack of access to investigations and consequent delay in diagnosis and treatment.

*‘Daughter not referred to hospital for a scan of her neck glands due to COVID-19 outbreak and hospitals not accepting any referrals from doctors.’ (UK)*

*‘They do not refer you to specialists, or do tests to assess and diagnose.’ (Spain)*

*‘The blood sampling did not work at all for two years.‘ (Sweden)*

Cancellation of services, delays in accessing health services and treatment were perceived to lead to increased severity of illness in their children or duration of illness.

*‘Diagnosis and treatment were delayed for weeks. She was in pain for weeks.’ (Spain)*

*‘Hard to get in touch and get an appointment, made my child more severely ill before we could get an appointment.’ (Sweden)*

*‘It took too long to get a diagnosis and treatment was delayed.’ (The Netherlands)*

*‘The delay in assessment delayed the diagnosis and favoured the progression of the illness.’ (Italy)*

*‘I truly believe if I didn’t have the medical know how or the treatments available and an understanding of how different drugs work that my daughter could have died due to poor NHS support during lockdown.’ (UK)*

Cancellation of services left parents either having to care for their child without access to healthcare or to seek alternative sources of help. In Sweden and Italy this included seeking private healthcare at a possible financial cost to parents. In the UK one parent reported serious consequences of a lack of access to inpatient mental health services:

*‘He should have been admitted and had a full and thorough evaluation and tapered treatment. Instead he was sent home, risked his life self-harmed and injured others, then instead of admitting him, they drugged him to almost sedation to help us to manage him during lockdown.’*

Parents also reported concerns that the cause of their children’s physical illness was not properly investigated leading them to question whether they had been prescribed the correct treatment:

*‘He may not have been prescribed the correct medication and the cause of his illness was not investigated’ (UK)*

*‘Antibiotics were prescribed without knowing if they were really necessary, to “play it safe.”’ (Spain)*

Difficulties in accessing non-urgent care were not reported in most parent’s reports of using hospital emergency services, although this varied between countries. In the Netherlands and the UK, some parents who took their child to emergency departments found these were unusually empty, reflecting the significant fall in paediatric hospital attendances and admissions during the lockdown [2, 7, 14, 15].

*‘Normally the waiting time at the Emergency Department is very long. However, now we were assessed and treated in 30 min. My child had less hours of pain, very positive!’ (The Netherlands)*

*‘Diagnosis and treatment was helpful and much quicker than usual because minor injuries unit was empty.’ (UK)*

Other parents reported delays when using hospital services, which they perceived to be related to insufficient staff or to surgery being postponed as the hospital was coping with an influx of people with COVID-19.

When parents were able to secure an appointment in primary care the appointments were frequently reported to be a telephone appointment leading to concerns of misdiagnosis *(‘Wrong initial diagnosis due to not making a face-to-face consultation’ Spain*) and delayed investigations, diagnosis and treatment, although other parents liked these appointments as they avoided face-to-face contact *(‘means we can still social distance’ UK*). Some parents from the Netherlands and the UK reported being able to send images of their child to the doctor either via email or an online platform which they found helpful (*‘they were fantastic, I sent over a photo via email so they didn’t have to see us’ UK*). Less common were reports of video consultations reflecting a low level of adoption of this technology in primary care in the UK [16].

When parents did have a face-to-face consultation for their child, some reported that the health professionals were afraid to examine their child or that the personal protective equipment made it difficult for them to examine their child. A few parents also reported that the personal protective equipment was frightening for their child, making the appointment an additionally stressful experience alongside the fear of contracting the virus.

#### Changes in parents’ help-seeking behaviours for a sick or injured child during pandemic lockdowns

Fear of being infected with COVID-19 was reported in all five countries and led some parents to delay in seeking help or to manage the illness or injury themselves when they would normally have sought medical help.

*‘Fear to take him to the hospital for fear of contagion’ (Spain)*

*‘We waited too long to seek medical help since we were anxious’ (The Netherlands)*

A ‘wait and see’ strategy was used by this parent:

‘*I waited to see if it would sort itself out before asking for him to be checked.*’ (UK)

Whilst others continued to seek help but worried about the risk of infection in doing so and some, in the UK, expressed concerns about not wanting to burden the already stressed health services.

#### The impact on parents of caring for a sick or injured child during lockdowns

Parents reported fear of contracting COVID-19 in any contacts with health services, which led to a fear of their child getting ill again and needing health care. Difficulties in accessing services and securing treatment for their child, real or anticipated, also led to a fear of needing health services.

*‘it has made us quite terrified of the children getting ill again and needing help which we feel would be difficult to get.’ (UK)*

*‘My young son suffers from gastroenteritis that requires admission in most cases. I was terrified that an episode of this type would happen.’ (Spain)*

Parents also worried about contracting COVID-19 themselves as they were concerned about how they would be able to care for their children:

*‘if I got sick who would look after my daughter with all my family shielding.’ (UK)*

Having to care for their child at home with limited access to services was reported to be stressful and as ‘a burden for us’ (Spain). Parents of children with complex or long term illness reported loss of the usual monitoring or continuing care for their child which left them feeling isolated:

*‘It was a feeling of extreme abandonment’. (Spain)*

Parents found it difficult to cope with their children’s illness when, not only was support from health services limited, but their social support was also reduced:

*‘It was very difficult to receive information alone, while worrying about your child’ (Sweden)*

*‘For parents of children with serious illnesses, confinement was a situation of extreme stress.’ (Spain)*

## Discussion

### Main findings

From an online survey disseminated through social media in five European countries, we have gained an understanding of parental help-seeking behaviour for their sick or injured child during the COVID-19 pandemic. In our international cohort consisting of 598 parents who completed the survey, parents were not deterred from seeking help during the COVID-19 lockdowns. This finding was similar for all the participating countries with their own healthcare system and level of restrictions in place. However, the percentage of parental help-seeking during lockdown was slightly lower than before lockdown in the UK. This might be explained by the period in which the survey was launched during the first lockdown in 2020 leading to an important decrease in paediatric emergency visits [2, 5]. The active survey periods in the other countries were during the second and third lockdowns and partly retrospective in Sweden. In these countries there was no evidence of a reduction in parental help-seeking during lockdown compared to before the lockdown. Parents experienced limited access to non-urgent care services leading to delay in diagnosis and treatment of their sick or injured child. Although parents sought help for their sick or injured child during lockdown, they reported that they were anxious and that the duration and severity of their child’s illness increased. The fear of getting infected with COVID-19 was reported in all five countries and in some cases led to delay in help-seeking. Perceived loss of support has been recognised in prior research to lead to increased anxiety in parents and increased perceived need for health services when their children are ill or injured [17, 18]. Parents were in social isolation as a consequence of lockdowns or stay-at-home advice, making the loss of support very real. In addition to this loss of support, there was considerable uncertainty experienced by parents, not only concerning their child’s illness or injury which is an inevitable part of parenting a sick child [19–22] but, during the pandemic, also concerning access to healthcare as there were unclear rules for health service use during lockdown. These stressors compounded the stress experienced by parents fearful of contracting the virus. The qualitative part of our study on parental experiences have shown what impact the COVID-19 pandemic had on parental stress and we believe that these insights could inform future strategies to improve access to healthcare during pandemics.

### Strengths and limitations

This study was carried out during the COVID-19 pandemic in five European countries using an online survey via social media enabling wide distribution of the survey. We collected data on help-seeking behaviour of parents living in different countries with their own changes to healthcare services during the COVID-19 pandemic. Extensive data were collected based on multiple choice answers, but parents were given the opportunity to elaborate on the chosen answers in the free text responses, which enabled thematic analysis to be performed and the provision of detailed quotes from parents. Some limitations of the study should be mentioned as well. First, the use of an online survey may raise concerns regarding generalizability [23]. The results may have limited generalizability due to selection bias since parents without access to social media or with limited literacy were not able to fill in the survey. However, nowadays almost all people living in the participating countries have a smartphone and have access to the internet [24–28]. Furthermore, the uptake was higher in areas where the researchers were located and local awareness of each of the institutions involved may have increased participants trust in the project. Secondly, respondent bias might be present when parents of children with more serious illness filled in the survey. Unfortunately, we could not calculate the response rate since the survey was distributed via virtual snowball sampling and only parents with a sick or injured child were eligible to fill in the survey. Additionally, we did not have data to track the dissemination process since different forms of social media and dissemination were used. Third, the largest group included children in the age group of five to twelve years, which might reflect the group of children in which the parents have time to fill in the survey. However, it does not reflect the age group of one to four years with highest incidence of medical attendees pre-pandemic [29]. Lastly, since the active survey period in most of the countries was after the pandemic restrictions were lifted, recall bias may also have influenced the results since parents have to recall the episode of their child’s illness or injury. However, this online survey enabled wide dissemination of the survey in five European countries and enabled data collection with great ease during the COVID-19 pandemic in which controlling the spread of COVID-19 was important [23].

### Implications for clinical practice

Parents from five European countries experienced limited access to non-urgent health services during the COVID-19 pandemic. However, a large European observational study on the impact of the COVID-19 pandemic showed that more severely ill children continued to visit the hospital more frequently than children with minor injuries or illnesses and that there was a higher reduction in children with low triage urgency [2]. Limited access to health services does not only have consequences for the disease severity and duration of the child’s illness, but may also have had consequences for usual care including vaccination programs in children and adolescents. Decreased vaccination rates or a delay in vaccination during the COVID-19 pandemic are reported worldwide [30]. As parental help-seeking behaviour and experiences during the COVID-19 pandemic are highlighted in this study, we would recommend healthcare professionals to provide professional safety netting advice and give parents explicit verbal and written information about when and where to seek help for their sick or injured child in the future [31]. It is important to spread public health messages at an early stage so that parents know when to visit medical services for their sick or injured child to mitigate delayed presentation. Parents also require information about any changes to access to health services, the safety systems in place to prevent attendees from contracting the virus and how to access support during any future lockdowns. Such information is required to address parental anxiety evidenced within this study.

## Conclusions

Our study shows that parents from five European countries with each country having its own healthcare system and restrictions during lockdowns, were not deterred from seeking help for their sick or injured child during the COVID-19 pandemic. Parents reported limited access to non-urgent services and were anxious about either their child or themselves catching COVID-19. This insight into parental perspectives could inform future strategies to improve access to healthcare, and to provide parents with adequate information concerning when and where to seek help and support during pandemics.

## Supplementary Information

**Additional file 1.**

## Data Availability

The datasets used and analyzed during the current study are available from the corresponding author on reasonable request.

## Abbreviations

COVID-19: Coronavirus Disease 2019
UK: United Kingdom
